# Evaluation of the Relationship between Current Internal ^137^Cs Exposure in Residents and Soil Contamination West of Chernobyl in Northern Ukraine

**DOI:** 10.1371/journal.pone.0139007

**Published:** 2015-09-24

**Authors:** Yuko Kimura, Yuka Okubo, Naomi Hayashida, Jumpei Takahashi, Alexander Gutevich, Sergiy Chorniy, Takashi Kudo, Noboru Takamura

**Affiliations:** 1 Department of Global Health, Medicine and Welfare, Atomic Bomb Disease Institute, Nagasaki University Graduate School of Biomedical Sciences, Nagasaki, Japan; 2 Division of Strategic Collaborative Research and 3Department of Isotope Medicine, Atomic Bomb Disease Institute, Nagasaki University Graduate School of Biomedical Sciences, Nagasaki, Japan; 3 Center for International Collaborative Research, Nagasaki University, Nagasaki, Japan; 4 Zhitomir Inter-Area Medical Diagnostic Center, Korosten, Ukraine; 5 Department of Isotope Medicine, Atomic Bomb Disease Institute, Nagasaki University Graduate School of Biomedical Sciences, Nagasaki, Japan; University of South Carolina, UNITED STATES

## Abstract

After the Chernobyl Nuclear Power Plant accident, the residents living around the Chernobyl were revealed to have been internally exposed to ^137^Cs through the intake of contaminated local foods. To evaluate the current situation of internal ^137^Cs exposure and the relationship between the ^137^Cs soil contamination and internal exposure in residents, we investigated the ^137^Cs body burden in residents who were living in 10 selected cities from the northern part of the Zhitomir region, Ukraine, and collected soil samples from three family farms and wild forests of each city to measured ^137^Cs concentrations. The total number of study participants was 36,862, of which 68.9% of them were female. After 2010, the annual effective doses were less than 0.1 mSv in over 90% of the residents. The ^137^Cs body burden was significantly higher in autumn than other seasons (p < 0.001) and in residents living in more contaminated areas (p < 0.001). We also found a significant correlation between the proportion of residents in each city with an estimated annual exposure dose exceeding 0.1 mSv and ^137^Cs concentration of soil samples from family farms (r = 0.828, p = 0.003). In conclusion, more than 25 years after the Chernobyl accident, the internal exposure doses to residents living in contaminated areas of northern Ukraine is limited but still related to ^137^Cs soil contamination. Furthermore, the consumption of local foods is considered to be the cause of internal exposure.

## Introduction

The Chernobyl Nuclear Power Plant (CNPP) accident on 26 April 1986 became the most serious nuclear power plant accident in our history. A large amount of radionuclides was released into the atmosphere over a period of 10 days following the accident. As a result of this accident, Ukraine, the Republic of Belarus and the Russian Federation were contaminated (in total, 150,000 km^2^ with more than 5 million inhabitants) [1]. As for Ukraine, the contamination with radionuclides was severe in the regions to the northwest and west of the power plant site, especially in the Kiev, Zhitomir and Rivne regions.

Following the first few weeks after the accident, radio-iodine such as ^131^I was the main contributor to the radiation exposures. Fallout of ^131^I caused a large thyroid internal exposure in inhabitants through inhalation and ingestion of contaminated foods. This is due to the fact that most of the radio-iodine taken into the body was accumulated in the thyroid gland. For the residents of the contaminated areas of the former Soviet Union who were not evacuated, the average thyroid dose was about 100 mGy [1]. The thyroid doses in pre-school children was some two to four times greater than the population average and caused a very large increase of thyroid cancer in the young population [2,3]. According to the United Nations Scientific Committee on the Effects of Atomic Radiation (UNSCEAR), 6,848 thyroid cancer cases were reported between 1991 and 2005 in the three countries (that is, in the whole of Belarus and Ukraine and in the four most contaminated regions of the Russian Federation) among children or adolescents under the age of 18 years at the time of the accident [1].

After the decay of ^131^I, radioisotopes of cesium (i.e. ^134^Cs and ^137^Cs) became the biggest factor in internal and external radiation exposure among inhabitants. Especially, ^137^Cs has caused continuous contamination due to its long half- life of 30 years [4]. Soil contamination with ^137^Cs led to the contamination of local foods from kitchen gardens and forests and caused internal exposure. Several studies reported that the relationship between whole body ^137^Cs concentration and the concentration in soil and local foods the residents consumed [5–7].

More than 25 years have passed since the accident, and internal exposure doses in the population have been decreasing due to the physical decay of ^137^Cs and changes in dietary patterns in the contaminated area [7–9]. Bernhardsson et al. reported the evaluation of internal and external exposure of inhabitants living in the Bryansk region of Russia in 1990–2008 [10]. They observed that, in 2008, the average effective dose (sum of external and internal exposure dose) from Chernobyl ^137^Cs to the residents was estimated to be 0.3 mSv^-y^, which corresponds to 8% and 1% of the estimated annual dose in 1990 and 1986, respectively. However, they also indicated that the effective dose from internal exposure is becoming increasingly important as the body burden of Chernobyl ^137^Cs, which is decreasing more slowly than the external exposure. Additionally, in our previous study, ^137^Cs still has been detected in one third of the residents living in the contaminated area of Ukraine, although the level of internal exposure dose in the residents is very low [9]. Even though the dose is limited, chronic internal exposure has continued, and it is unclear whether the ^137^Cs soil contamination level still relates to the internal exposure in residents. Therefore, we conducted the present study to evaluate the current situation of internal exposure dose due to ^137^Cs in residents living in northern Ukraine and to evaluate the relationship between soil contamination levels of ^137^Cs and internal exposure in residents.

## Material and Methods

The study protocol was approved by the Institutional Review Board of Korosten Inter-Area Medical Diagnostic Center (No.002) and the ethical committee of Nagasaki University Graduate School of Biomedical Sciences (No.12122865). Also, the study was approved for the collection of soil samples by the regional office of Zhitomir region in Ukraine.

### Study area

**Fig 1:**
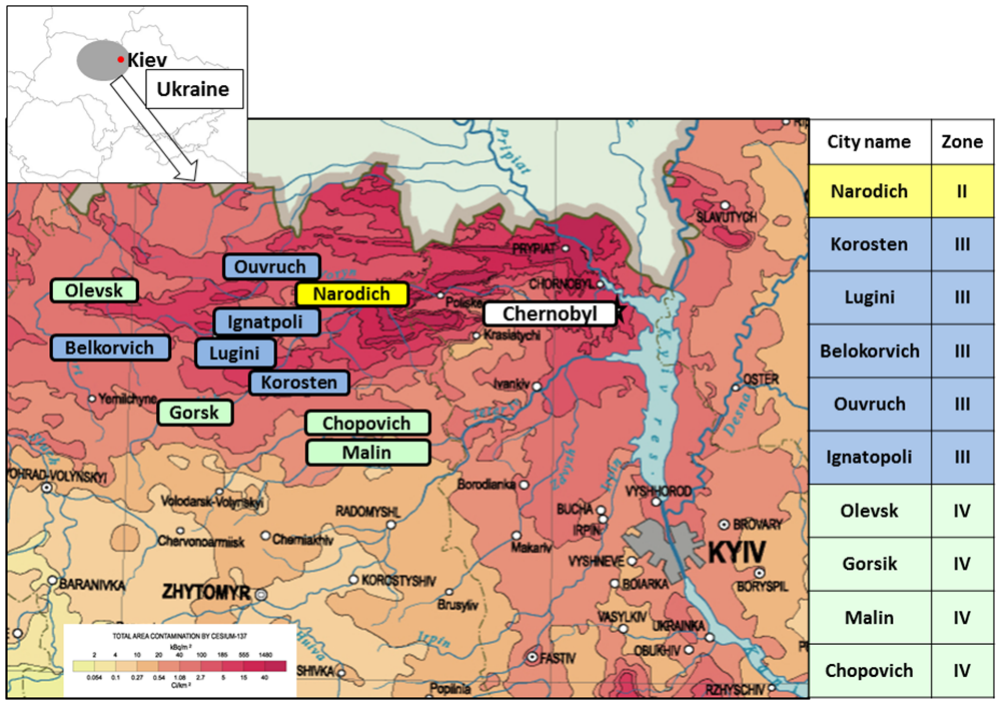
The study area. 10 cities were selected from north part of Zhitomir region, Ukraine, southwest from Chernobyl. In Ukraine, a classification of 4 contamination zones was established. The zones were defined according to soil contamination levels of ^137^Cs as zone I (> 1480 kBq/m^2^), zone II (555 kBq/m^2^–1480 kBq/m^2^), zone III (185 kBq/m^2^–555 kBq/m^2^) and zone IV (37 kBq/m^2^–185 kBq/m^2^). The 10 cities we chosen were classified to zone II-IV as shown in the table. This map was modified from copyright free maps provided by DesignExchange Co. Ltd and Ministry of Emergency and Protection of Chernobyl-affected people, Ukraine.

We conducted this study in the northern part of the Zhitomir region in Ukraine. This area is located southwest of Chernobyl and was strongly affected by the accident at CNPP. From this region, we selected 10 cities with 100 or more inhabitants who underwent ^137^Cs body burden screening at the Zhitomir Inter-Regional Diagnostic Center (referred to hereafter as “the Center”) each year in Korosten city (Fig 1). In Ukraine, a classification of four contamination zones was established. The zones were defined according to soil contamination levels of ^137^Cs as zone I (> 1480 kBq/m^2^), zone II (555 kBq/m^2^–1480 kBq/m^2^), zone III (185 kBq/m^2^–555 kBq/m^2^) and zone IV (37 kBq/m^2^–185 kBq/m^2^). The 10 cities we chose were classified to zone II, III and IV, as shown in Fig 1. Narodichi was the only city in zone II for which the Center had responsibility for health screening and which had around 100 residents undergoing WBC screening each year. Data for the ^137^Cs body burden of residents in zone I were not available, as the Center did not cover the health care screening of residents living there.

### Measurement of ^137^Cs body burden

We investigated the ^137^Cs body burdens of residents who were living in these 10 cities and visited the Zhitomir Inter-Regional Diagnostic Center in Korosten city for regular health screening from January 2009 to December 2012 (S1 Data). The ^137^Cs body burdens in the inhabitants were measured for 2-minutes using a whole body counter (WBC), a γ-spectrometer, model 101, equipped with a collimator (Aloka Co., Ltd.). The counter was equipped with a 7.6 × 7.6 × 7.6 cm NaI (TI) detector with a 5-cm-thick lead shield. The Covell method was used for computation of total absorption peak. The participants sat on a sliding chair and the height and angle of the detector were adjusted to make the participant’s abdomen face it. The back and the seat of the chair were shielded with lead plates. Gamma rays emitted from the body were counted by the detector and analyzed with a 240-channel spectrometer. The minimum detectable ^137^Cs body burden was 270 Bq/body. The counter was calibrated once a year, using a phantom. Participants who did not have any ^137^Cs exposure were considered to be “0 Bq.” In accordance with the manufacture’s instruction, ^137^Cs body burden was calculated and the obtained values were corrected for body weight. We calculated concentration by body weight (Bq/kg). Furthermore, the annual exposure dose was estimated based on the effective dose coefficient of 2.5 × 10^−3^ mSvy^-1^ / Bq kg^-1^ [6].

### Measurement of ^137^Cs in soil

For evaluating current soil contamination, we collected soil samples (soil depths of 0–5 cm) from three family farms and wild forests of each city from April to May, 2013. Before collecting soil samples in forest areas, we removed fallen leaves and withered grass covering the soil surface. All samples were dried for 24 hours at 105°C and were sieved to remove pebbles and organic materials (<2 mm) before measuring radioactivity. After preparation, the soil samples were put into plastic containers made of polypropylene and analyzed with a high purity germanium detector (ORTEC®, GMX30-70, Ortec International Inc., Oak Ridge, TN, USA) coupled to a multi-channel analyzer (MCA7600, Seiko EG&G Co., Ltd., Chiba, Japan) for 80,000 seconds. The measuring time was set to detect the objective radionuclide, and the gamma-ray peaks used for the measurements were 604.66 keV for ^134^Cs (2.1 y) and 661.64 keV for ^137^Cs (30 y). Decay corrections were made based on the sampling data, and detector efficiency calibration for different measurement geometries was performed using mixed activity standard volume sources (Japan Radioisotope Association, Tokyo, Japan).

### Statistical analysis

Data is expressed as a median (25^th^–75^th^ percentile) or mean (SD). Annual exposure dose and ^137^Cs body burden were contained 0 and distributed in a skewed manner. Therefore, we added 1 to those values and a logarithmic transformation was performed for the analysis. To assess the seasonal effect, the measurement periods were divided into four seasons (i.e., March–May for spring, June–August for summer, September–November for autumn, December–February for winter). We conducted a one-way analysis of variance (ANOVA) to evaluate the differences in the ^137^Cs body burdens in each season and each zone. In addition, we compared the ^137^Cs body burdens in each zone using an analysis of covariance (ANCOVA) adjusted between the zones for age group (0–10 years, 11–20, 21–60, and 61+), sex, and year of examination.

The correlation between the ^137^Cs concentration in soil samples and the proportion of residents with an estimated annual exposure dose that exceeded 0.1 mSv was evaluated using the Pearson product-moment correlation coefficient. We used the estimated annual exposure dose exceeding 0.1 mSv in each city rather than median ^137^Cs body burden, because the median values of the ^137^Cs body burdens for each city (except for Lugini and Narodichi) were below the detectable level, although we intended to analyze the correlation between the ^137^Cs body burden and the ^137^Cs concentration in soil samples. Statistical analysis was performed using SPSS Statistics, v.22.0 for Mac (SPSS Japan, Tokyo, Japan). P values less than 0.05 were considered statistically significant.

## Results

The population, distribution of age, and outcome of ^137^Cs screening from 2009 to 2012 are shown in Table 1. The number of participants in each year was around 9,000 and almost 70% were female. The proportion of participants from zone IV was biggest in 2010 and smallest in 2009. The median ^137^Cs body burden was below the detectable level for the entire measurement period. The frequency of the residents who had a detected amount of ^137^Cs by WBC was from 14.7% to 44.9%. After 2010, the annual effective doses were less than 0.1 mSv in over 90% of the residents, and the number of residents that exceeded 1 mSv y^-1^ was less than 10. The median body burden of ^137^Cs in both males and females was below the detectable level, but significantly higher in males than in females (p < 0.001). The median body burden of ^137^Cs in all age categories was also below the detectable level; comparing categories, it was significantly lower in the 0–10 years age range and significantly higher in the 11–20 years range (p < 0.001).

**Table 1:** Population, age and outcome of ^137^Cs screening from 2009 to 2012.

	2009	2010	2011	2012	Total
Number of participants	9,257	8,206	9,637	9,762	36,862
	Number of participants in each zone (%)				
II	97 (1.1)	56 (0.7)	87 (0.9)	86 (0.9)	326 (0.9)
III	7,764 (83.9)	6,542 (79.7)	56 (0.7)	7,851 (80.4)	29,917 (81.2)
IV	1,396 (15.1)	1,608 (19.6)	1,790 (18.6)	1,825 (18.7)	6,619 (18.0)
Female (%)	65.0	73.4	68.2	69.5	68.9
Age (±SD)	39.0 (±19.3)	38.2 (±19.1)	41.0 (±18.7)	43.4 (±18.0)	40.5 (±18.9)
^137^Cs body burden (Bq/kg)	0.00	0.00	0.00	0.00	0.00
25th–75th	0.00–23.83	0.00–0.00	0.00–19.43	0.00–12.84	0.00–15.44
^137^Cs detected population (%)	44.9	14.7	41.3	30.7	33.5
< 0.1 mSv/year (%)	85.90	98.7	92.4	97.5	93.5
>1 mSv/year (n)	30	3	8	5	46

Age is expressed as the mean (SD) and ^137^Cs body burden is expressed as the median (25th–75th).

**Fig 2:**
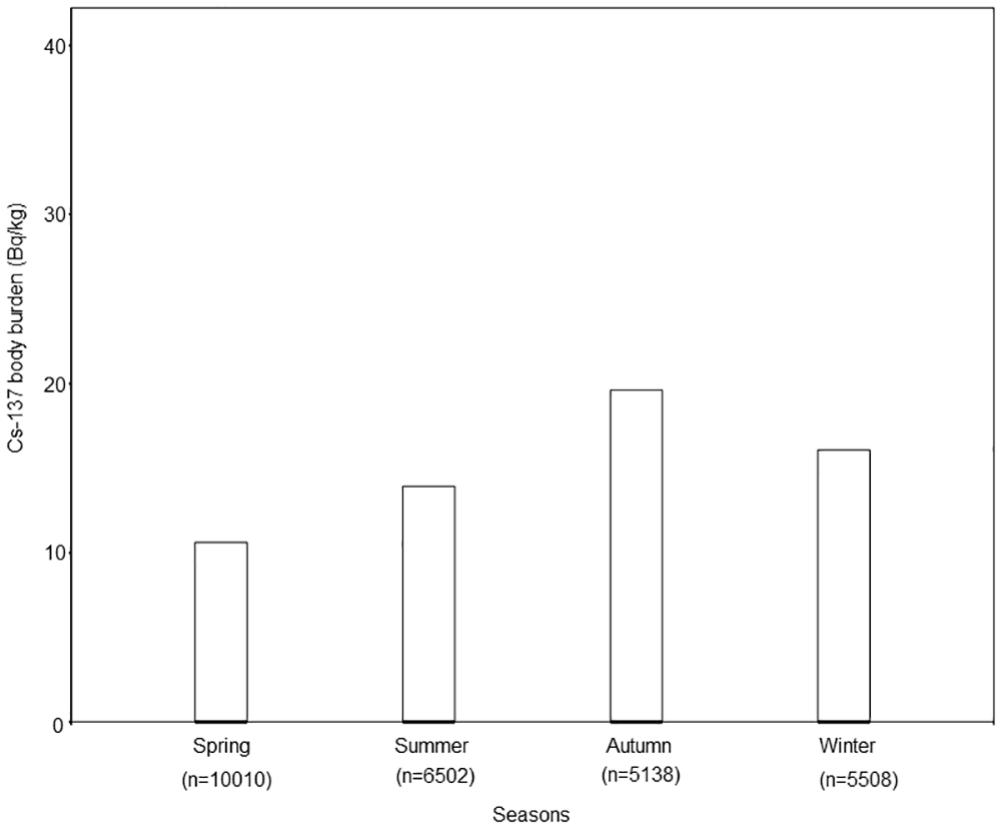
Seasonal difference of Cs-137 body burden in residents. The end of the box indicate the positions of the 75th percentiles of the data. The median Cs-137 body burden was below the detectable level in each season however significantly higher in autumn than other seasons (p<0.001).

Fig 2 shows the seasonal differences in ^137^Cs concentration between 2009 and 2012. Although median ^137^Cs body burden was below the detectable level in each season, ^137^Cs body burden in autumn was significantly higher than other seasons (p < 0.001). The frequency of residents with an annual dose that exceeded 0.1 mSv in each season was, from spring to winter, 26.3%, 29.4%, 43.2% and 35.0%, respectively.

Table 2 shows the results of screening by WBC in each city. In most cities, the median ^137^Cs body burden was below the detectable level. However, in Narodichi (zone II) and Lugini (zone III), the median body burdens of ^137^Cs were 16.31 Bq/kg and 10.50 Bq/kg, respectively. The proportion of residents with a dose of less than 0.1 mSv y^-1^ was more than 90%, except for these two cities.

**Table 2:** Total population and outcome of ^137^Cs screening in 10 chosen cities from 2009 to 2012.

	Narodichi	Korosten	Lugini	Belkorvich	Ouvruch	Ignatpoli	Gorsk	Olevsk	Malin	Chopovich
Zone	II	III	III	III	III	III	IV	IV	IV	IV
No.	326	25881	1529	749	1430	328	369	1705	4181	364
^137^Cs body burden (Bq/kg)	16.31	0	10.50	0	0	0	0	0	0	0
(25th–75th)	(0–39.74)	(0–15.33)	(0–24.06)	(0–19.85)	(0–9.60)	(0–19.83)	(0–0)	(0–19.19)	(0–0)	(0–0)
<0.1 mSv/y (%)	75.46	93.40	88.29	92.52	91.75	93.29	96.21	92.90	97.94	98.63

^137^Cs body burden is expressed as the median (25th–75th).

**Fig 3:**
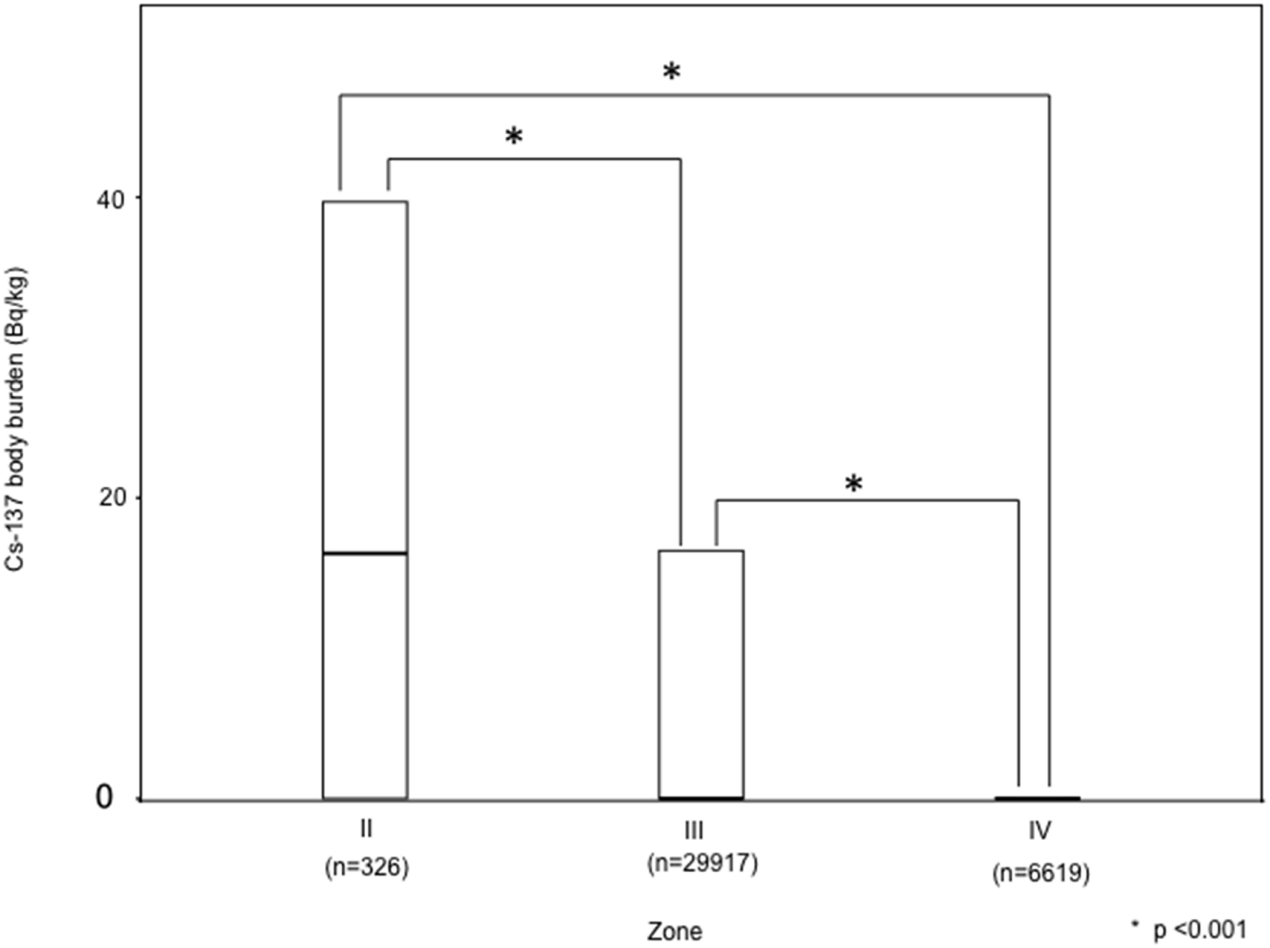
Cs-137 body burden in the residents living in each zone. The end of the box indicate the positions of the 75th percentiles of the data. The Cs-137 body burden was significantly higher in more contaminated zone.

Fig 3 shows the ^137^Cs body burden in residents living in each zone. The median ^137^Cs body burdens (25th–75th) were 16.31 Bq/kg (0–39.74) for zone II, 0 Bq/kg (0–16.28) for zone III, and 0 Bq/kg (0–0) for zone IV, respectively. The ^137^Cs body burden was significantly higher in more contaminated zones (p<0.001), where the highest concentration was in zone II and the lowest was in zone IV; with adjustments by age category, sex, and the year of examination, as shown in Table 3, significant differences between zones were still observed (p<0.001). The frequency of the residents who exceeded 0.1 mSv in each zone was also significantly higher in more contaminated zones (p < 0.001) (24.5% for zone II, 6.9% for zone III and 3.4% for zone IV).

**Table 3:** Log(^137^Cs body burden + 1) of each zone adjusted by age category, sex, and year of examination.

	Zone II	Zone III	Zone IV	P value
Not adjusted	0.98 ± 0.046	0.49 ± 0.004	0.34 ± 0.007	<0.001
Age category adjusted	0.98 ± 0.038	0.49 ± 0.004	0.35 ± 0.008	<0.001
Sex adjusted	1.00 ± 0.038	0.49 ± 0.004	0.35 ± 0.008	<0.001
Year of examination adjusted	0.98 ± 0.038	0.49 ± 0.004	0.34 ± 0.008	<0.001
Age category, sex, and year of examination adjusted	1.00 ± 0.037	0.49 ± 0.004	0.36 ± 0.008	<0.001

The ^137^Cs body burden was significantly higher in more contaminated zones (p<0.001), with adjustments by age category, sex, and year of examination, significant differences between zones were still observed (p<0.001). The Log(^137^Cs137 body burden + 1) is expressed as the mean ± standard error.

We also analyzed seasonal change in each zone (Table 4). In zone III and zone IV, the median body burden of ^137^Cs was below the detectable level, but the ^137^Cs body burden increased in autumn and winter, and weak but significant seasonal change was observed (p < 0.001 for zone III and zone IV). On the other hand, no significant seasonal change of ^137^Cs body burden was observed in zone II (p = 0.959). However, as the ^137^Cs body burden in Lugini was as high as that in Narodich, a zone II city, we divided 10 cities into two groups: Narodichi and Lugini as the highly contaminated area group and the other eight cities as the not highly contaminated area group. We evaluated the seasonal changes in both area groups, and significant seasonal changes were observed (Table 4).

**Table 4:** Seasonal differences in the ^137^Cs body burdens of each zone: the highly contaminated area and the not highly contaminated area.

	Spring	Summer	Autumn	Winter	P value
Zone					
II	16.10 (0–32.05)	16.55 (0–39.46)	16.45 (0–45.00)	15.63 (0–45.00)	0.959
III	0 (0–11.03)	0 (0–15.00)	0 (0–21.10)	0 (0–16.79)	<0.001
IV	0 (0–0)	0 (0–0)	0 (0–12.59)	0 (0–12.66)	<0.001
Highly contaminated area vs. not highly contaminated area					
Highly	0 (0–21.16)	0 (0–22.84)	16.33 (0–34,54)	12.83 (0–25.11)	<0.001
Not highly	0 (0–0.02)	0 (0–13.57)	0 (0–18.94)	0 (0–15.62)	<0.001

In zone III and zone IV, weak but significant seasonal changes were observed. On the other hand, no significant seasonal change in the ^137^Cs body burden was observed in zone II. However, we evaluated the seasonal changes in the highly contaminated area group, comprised of Narodichi and Lugini, and in the not highly contaminated area group, comprising the other eight cities, separately, and in both area groups, significant seasonal changes were observed. The ^137^Cs body burden is expressed as the median (25th–75th).

**Fig 4:**
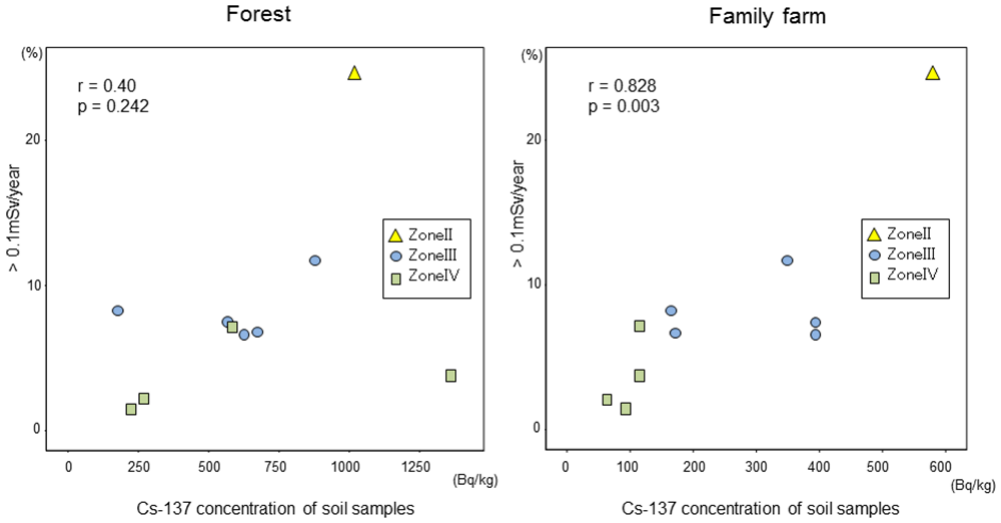
Correlation between mean ^137^Cs concentration of soil samples and rate of population with dose of >0.1mSv/year of each city.

Fig 4 shows the relationship between mean ^137^Cs concentrations of soil samples collected at forest and family farms, and the proportion of residents with estimated annual exposure dose exceeded 0.1 mSv in each city. Concentrations of ^137^Cs tended to be higher in soil samples from contaminated areas. There was a significant correlation between ^137^Cs concentration in soil samples from family farms and the proportion of residents with estimated annual exposure dose that exceeded 0.1 mSv in each city (r = 0.828, p = 0.003). On the other hand, we found the tendency of increase of prevalence of estimated annual exposure dose that exceeded 0.1 mSv in each city in proportion as increasing the ^137^Cs concentrations in soil samples from forests, but the relationship not significant (r = 0.40, p = 0.242).

## Discussion

In the present study, we evaluated the current situation of internal ^137^Cs exposure to the residents and the relationship between soil contamination levels of ^137^Cs and internal exposure in the contaminated area of northern Ukraine.

Recently, we conducted screening for whole body ^137^Cs concentrations in residents living in the Zhitomir region from September 1996 to August 2008 [9], and showed that ^137^Cs body burden is continuously decreasing in the area. In particular, after 2003, the annual effective dose decreased to 0.1 mSv y^-1^ for 95% of the residents, and more than half of the residents had internal exposure doses below the detectable level. In the present study, we showed that, although ^137^Cs was still detected in more than 30% of residents living in 10 cities from 2009 to 2012, the estimated annual exposure doses were below 0.1 mSv in more than 90% of the residents, and that the number of residents with doses that exceeded 1 mSv y^-1^ (ICRP dose limit for the general public [11]) after 2010 was less than 10. As shown in Fig 1, the northern part of Zhitomir extends southwest from the CNPP, and it was reported as the most contaminated area in northern Ukraine [4]. In the present study, we selected relatively big cities from each zone in this area. In particular, Narodichi is the biggest zone II city where people live in northern Ukraine. Therefore, regarding the levels of ^137^Cs contamination, we considered that the participants living in this study area could represent northern Ukraine, and the results suggest that currently, the internal exposure dose to residents due to ^137^Cs has been limited in northern Ukraine. In Table 1, the percentage of the population affected by ^137^Cs had drastically decreased in 2010, increasing again in 2011. The percentage of the population from zone IV (in whole participants each year) was biggest in 2010 and smallest in 2009. We suppose that this difference may have caused the increased internal exposure of residents in 2010.

As previous studies reported, the concentration of ^137^Cs was higher in males than in females. This difference is likely due to body size and composition. Semioshkina *et al*. showed that ^137^Cs is highly transferred to the spleen, lungs, heart, muscles, kidneys, skin and bone in horse tissue taken on the 90^th^ day after the beginning of radionuclide administration [12]. Because ^137^Cs accumulates in muscle and bone, the amount of ^137^Cs body burden changes depending on the amount of muscles in one’s body. For the same reason, the ^137^Cs body burden was considered to be lowest in the age category 0–10 years because of children’s lower muscle mass.

Previous studies conducted 5–10 years after the Chernobyl accident revealed that there was a close relationship between whole body ^137^Cs concentrations and radioactivity in the soil [5,6]. Takatsuji *et al*. measured the radioactivity in soil and child food samples from farms near Klincy in Russia (59–270 GBq km^-2^ ^137^Cs), Mogilev (56–270 gBqkm^-2^ ^137^Cs) and Gomel (36–810 GBq km^-2^ ^137^Cs) in Belarus [5]. They found that there was a significant correlation between the ^137^Cs body burden and soil radioactivity, and also the average contamination level of their current residence. In the present study, we also observed that the median ^137^Cs body burden was significantly higher in the residents living in the cities classified as more contaminated, and a positive relationship between ^137^Cs concentrations in the soil samples of family farms and the frequency of residents with annual exposed dose exceeded 0.1 mSv. These results suggest that the soil contamination level is still related to internal exposure due to ^137^Cs in residents. In Lugini, however, the ^137^Cs body burden was similar to that in Narodichi, although it is in zone II. Lugini is located closer to zone II than other cities in zone III, and a small part of Lugini is actually in zone II. Nevertheless, we designated Lugini as zone III because most of Lugini is classified as zone III. This high ^137^Cs body burden in Lugini might be explained by the possibility that examinees tend to be from a highly contaminated area of Lugini.

In addition, we found a weak, but significant, seasonal change of ^137^Cs body burden in residents. Several studies have reported the seasonal difference in ^137^Cs concentration due to the change in diet [6,8]. Handl *et al*. investigated the ^137^Cs daily intake of residents living in zone II, north Ukraine, and found that all agricultural products such as potatoes, turnips and cabbage showed low ^137^Cs concentrations between 0.2 and 20 Bq kg^-1^ fresh weight, whereas wild berries and mushrooms showed high ^137^Cs concentrations of 2,600 Bq kg^-1^ dry weight and 200,000 Bq kg^-1^ dry weight, respectively [13]. Travnikova *et al*. also reported that in the Bryansk region of Russia, the contribution of natural products to internal exposure increased from 6% in 1987 to 25% in 1996 [7]. As shown in Fig 2, in our study, ^137^Cs body burden was the highest in autumn. In addition, this seasonal change was observed in both highly contaminated and not highly contaminated area residents. This suggests that the increased consumption of natural products from contaminated forests largely contributed to the increases in ^137^Cs concentrations in autumn and winter among the residents living in not only the highly contaminated area but also the relatively lower contaminated area, even 30 years after the accident.

The Fukushima Dai-ichi Nuclear Power Plant (FNPP) accident occurred following the Great East Japan Earthquake on 11 March 2011, and a large area was contaminated by radionuclides, including ^134^Cs and ^137^Cs emitted from the plant [14,15]. Based on the experiences of the CNPP accident, monitoring of food and drinking water by Japanese and prefectural governments began on 16 March 2011. Selected foodstuffs (e.g., milk, vegetables, grains, meat, fish) containing radioactive material exceeding the provisional regulation values recommended on 17 March 2011 by Japan’s Ministry of Health, Labour and Welfare were prohibited from distribution on 21 March 2011 and from consumption on 23 March 2011. As a consequence of this strategy, internal exposure to ^134^Cs and ^137^Cs among residents of Fukushima has been very limited [16–18]. On the other hand, our study shows that the ^137^Cs body burden in residents living in the contaminated area still correlates with the ^137^Cs soil contamination level of their family farm even now, almost 30 years after the nuclear power plant accident. This suggests that long-term food control and decontamination of farmland will be effective in minimizing internal radiation exposure among residents of Fukushima.

There are several limitations in this study. We evaluated soil contamination only from 0–5 cm in depth. A recent analysis of soil contamination in Masany, Belarus (which is 8km from the Chernobyl Nuclear Power Plant) reported that the concentration of detected ^137^Cs was higher in surface soil (0–5cm) samples than in lower layers (5–10cm), and that prevalent radionuclides had accumulated mainly in the surface layer [19]. With regard to samples from the family farm, the residents have been cultivating their farm for many years to a depth of more than 20 cm with a hoe and farm tractor, and so the contamination level of soil should be the same as the depth at which crops can take root and grow. For this reason, we consider that surface soil is representative of any soil contamination that may cause food contamination. We should acknowledge the possibility of the duplication of study participants. Participants could visit the center to take their annual health screening, so certain participants might have undergone WBC screening more than twice between 2009 and 2012. However, in consideration of the biological half-life of ^137^Cs, we considered that the data of each participant’s ^137^Cs body burden in each year could be counted per respective participant. Additionally, there might be a bias in the selection of study participants, as the subjects of this study were the residents who visited the center and underwent WBC screening in each year. Therefore, we need to carefully evaluate whether our data could represent all the residents of each area. We also did not conduct interviews to establish participants’ period of residence at their present home, or their lifestyle and dietary habits. Furthermore, we could not measure the ^137^Cs concentration of products from family farms and forests and could not evaluate the dietary habits of residents.

In conclusion, more than 25 years after the Chernobyl accident, the internal exposure doses in residents living in contaminated areas of the northern Zhitomir region in Ukraine are limited but still related to ^137^Cs soil contamination. Additionally, the consumption of local foods is considered to be the cause of internal exposure. Unnecessary radiation exposure should be avoided even though the exposure dose is limited. Therefore, further studies are required to clarify the accurate mechanism of internal exposure in residents to provide useful data for risk communication.

## Supporting Information

S1 Data Raw data of ^137^Cs body burden measured by WBC. (XLSX)
